# Planetary Health in Health Guidelines and Health Technology Assessments: A Scoping Review

**DOI:** 10.1002/gin2.70056

**Published:** 2025-12-13

**Authors:** Pakeezah Saadat, Maheen Raja, Andrea J. Darzi, Charlotte T. J. Michels, Alina Herrmann, Karolina Anna Scahill, Laura M. Jewell, K. M. Saif‐Ur‐Rahman, Hendrik Napierala, Ruben Heuer, Rebecca L. Morgan, Grigorios I. Leontiadis, Ignacio Neumann, Fiona A. Miller, Holger J. Schünemann, Thomas Piggott

**Affiliations:** ^1^ Department of Health Research Methods, Evidence, and Impact McMaster University Hamilton Canada; ^2^ Department of Anesthesia McMaster University Hamilton Canada; ^3^ Knowledge Institute of the Dutch Association of Medical Specialists Utrecht the Netherlands; ^4^ Institute of Global Health Medical Faculty Heidelberg University, Heidelberg University Hospital Heidelberg Germany; ^5^ Institute of General Medicine Medical Faculty Cologne University, Cologne University Hospital Cologne Germany; ^6^ College of Medicine and Veterinary Medicine University of Edinburgh Edinburgh UK; ^7^ Evidensia Sodra Djursjukhuset Kungens Kurva Kungens Kurva Sweden; ^8^ Department of Family Medicine Queens University Kingston Canada; ^9^ College of Medicine, Nursing and Health Sciences University of Galway Galway Ireland; ^10^ Evidence Synthesis Ireland and Cochrane Ireland University of Galway Galway Ireland; ^11^ Institute of General Practice and Family Medicine Charite ‐ Universitatsmedizin Berlin, corporate member of Freie Universitat Berlin and Humboldt Universitat zu Berlin Berlin; ^12^ Division of Evidence‐Based Medicine, Department of Dermatology, Venereology and Allergology Charite ‐ Universitatsmedizin Berlin, corporate member of Freie Universitat Berlin und Humboldt Universitat zu Berlin Germany; ^13^ School of Medicine Case Western Reserve University Cleveland Ohio USA; ^14^ Department of Medicine McMaster University Hamilton Canada; ^15^ School of Medicine Universidad San Sebastian Santiago Chile; ^16^ GRADE Conosur Universidad San Sebastian Santiago Chile; ^17^ Institute of Health Policy, Management & Evaluation, Dalla Lana School of Public Health; Collaborative Centre for Climate, Health & Sustainable Care University of Toronto Toronto Canada; ^18^ Clinical Epidemiology and Research Center (CERC) Humanitas University and IRCCS Humanitas Research Hospital Milano Italy

**Keywords:** GRADE, health guidelines, health technology assessments, planetary boundaries, planetary health

## Abstract

**Introduction:**

Individual human health is inextricably linked to planetary health, which refers to the health of human civilization and the natural systems on which it depends. These systems are captured by the nine planetary boundaries which together define a safe operating space for humanity. The health sector contributes to the transgression of these boundaries, thereby jeopardizing both individual and planetary health. However, there is a growing interest in the health sector in incorporating the preservation of planetary boundaries into health guidelines and health technology assessments (HTAs), which synthesize evidence and guide healthcare decisions.

**Methods:**

This scoping review aims to describe methodological guidance and considerations for incorporating planetary health in health guidelines and HTAs. We conducted a scoping review adhering to the JBI methodology including articles from several databases since inception until September 2023. We used narrative synthesis and descriptive statistics to describe eligible studies.

**Results:**

Of the 38 included studies, 14 (37%) were commentaries, six (16%) were methodological papers, and four (10·5%) were health guidelines. Included studies focused primarily on greenhouse gas emissions as an outcome. The included health guidelines were rated at a median of 52% (range: 16% to 67%) on AGREE II for methodological quality.

**Discussion:**

Key findings pertain to the normative significance of planetary health in guidelines and HTAs, scope and quality of included studies, inconsistencies in methods, and challenges in implementation. These findings will inform future Grading of Recommendations, Assessment, Development, and Evaluations (GRADE) guidance on planetary health.

**Registration:**

A protocol of this scoping review was registered in Open Science Framework (https://osf.io/3jmsa) and published in the journal Systematic Reviews (10.1186/s13643‐024‐02577‐2).

## Summary

- Planetary Health is rarely integrated into health guidelines and HTAs; only four guidelines and no HTAs explicitly considered it during the guideline development process.
- Existing work focuses entirely on GHG emissions, with limited attention paid to other planetary boundary outcomes, such as pollution, freshwater change, and biodiversity.
- Methodological approaches are inconsistent; only one guideline used GRADE, and guideline panels rarely included planetary health or related field experts.
- There is an urgent need for standardized frameworks and guidance to systematically incorporate planetary health into future guidelines and HTAs.

**Abbreviations**
AGREE II: Appraisal of Guidelines for Research and Evaluation II
EtD: evidence to decision
GHG: Greenhouse Gas
GRADE: Grading of Recommendations, Assessment, Development, and Evaluations
HTA: health technology assessments
IPAC: infection prevention and control
LCA: life cycle assessments

## 1 Background

Methodological developments in health guidelines and health technology assessments (HTAs) have significantly advanced health decision‐making over the last few decades. However, improvements in health outcomes and healthcare delivery have also contributed to the exploitation of our planet's limited resources, resulting in transgression of planetary boundaries—‘processes that are critical for maintaining the stability and resilience of Earth system as a whole’ [1, 2]. These unintended consequences have exacerbated climate change and undermined planetary health. If the global health system were a country, it would rank fifth in greenhouse gas (GHG) emissions after China, the United States, India and Russia. Health system emissions account for 4.6% of global GHGs [3], up to 5% and 10% of GHGs in Canada [4] and the United States [5], respectively [6]. GHG emissions fall under three scopes according to the Greenhouse Gas Protocol [7]: (1) direct emissions from healthcare facilities, (2) indirect emissions, such as excessive electricity use and (3) other indirect emissions from upstream and downstream supply chains.

In recognition of the complex ways in which the health sector both affects and is affected by global environmental crises, there is increasing interest in ‘planetary health’—a field concerned with the health of human civilization and the natural systems it depends on—as evidenced by new journals and burgeoning research [8]. While environmental crises in general have received substantial scientific attention, minimal attention has been paid to integrating planetary health considerations in health guideline and HTA processes. This oversight in health systems' approach to planetary health fails to recognize the interconnectedness between human health, animal health, and the health of our planet [9]. As environmental crises accelerate, considering planetary health in health guidelines and HTAs is becoming increasingly urgent [10].

The Grading of Recommendations, Assessment, Development, and Evaluations (GRADE) methodology is increasingly being used in guideline development. This tool serves as a robust framework for transitioning from evidence to establishing recommendations [11]. The GRADE approach for developing recommendations using the evidence to decision (EtD) framework addresses a broad range of criteria including benefits, harms, balance of effects, certainty of evidence, resources required, cost‐effectiveness, equity, acceptability, and feasibility and allows for flexibility in tailoring decision criteria [12, 13]. However, the current EtD criteria do not explicitly prompt the consideration of planetary health.

In response, the GRADE Working Group established a new subgroup on planetary health to enhance its integration into health guidelines. As a preliminary assessment, our group revisited the recommendation of red meat consumption to ‘continue current consumption of both unprocessed and processed red meat’ and suggested that, under planetary health considerations, this recommendation would likely shift toward reducing red meat consumption [14]. This reassessment underscores the potential for planetary health considerations to reshape guideline recommendations, highlighting the need for a more structured approach to systematically incorporate planetary health into health decision‐making.

By addressing planetary health, guideline developers and HTA reporters will be better equipped to assess the impacts of health interventions more comprehensively on both humans and the planet. It may encourage the mitigation of harmful healthcare practices, such as the overuse of single‐use plastics [15], carbon‐intensive transport [16], or the use of facemasks [17]. This scoping review aims to identify the extent to which planetary health considerations are incorporated in current health guidelines and HTAs, ultimately informing the development of future GRADE guidance for integrating planetary health into decision‐making [18].

## 2 Review Questions

Our objective was to address the following questions:

1. What dimensions of planetary health have been considered in health guidelines and HTAs?
2. How have guideline development methodologies suggested that health guidelines or HTAs consider the environment, climate change, or planetary health?
3. What methods have health guidelines and HTAs used to incorporate evidence and assess the certainty of evidence regarding planetary health outcomes?

## 3 Methods

This scoping review was conducted in accordance with the JBI (formerly Joanna Briggs Institute) methodology for scoping reviews [19, 20] and reported as per the PRISMA‐ScR [21] (Appendix S1). Recent updates of PRISMA 2020 [22], particularly the scoping review modifications outlined in the scoping review chapter of the JBI manual on evidence synthesis [23], were also taken into consideration. We registered our protocol in Open Science Framework (https://osf.io/3jmsa) and published it in the journal Systematic Reviews [24].

### 3.1 Eligibility Criteria

#### 3.1.1 Participants

This criterion is not relevant to this scoping review, as we investigated methods and approaches for integrating planetary health considerations in health guidelines and HTAs targeting human populations. Guidelines for animal health or other nonhuman populations were not included in this review.

### 3.2 Concept

According to *The Lancet*, planetary health is defined as ‘The achievement of the highest attainable standard of health, wellbeing, and equity worldwide through judicious attention to the human systems—political, economic, and social—that shape the future of humanity and the Earth's natural systems that define the safe environmental limits within which humanity can flourish. Put simply, planetary health is the health of human civilisation and the state of the natural systems on which it depends’ [8]. The original concept of ‘planetary health’ as defined by Whitmee et al. takes a human‐centered approach in describing the interconnections between the environment, ecosystems, animal and human health [9, 25]. Yet, the discourse on planetary health partly shifts its focal point towards outcomes that transcend human‐centric considerations. For instance, the ‘planetary health check’ published in 2024 provides information about the state of the Earth based on its planetary boundaries, not considering any direct or indirect human health outcomes [26]. In our scoping review, we focus on outcomes based on planetary boundaries, as these represent an important addition to the direct human health outcomes that have traditionally been the focus of health guidelines and HTAs. Ultimately, there is a growing imperative for health guidelines and HTAs to place substantial emphasis on the equilibrium of natural systems, as measured through the planetary boundaries concept, recognizing that the preservation of human health is fundamentally interconnected with these processes [2, 27, 28].

The definition of health guidelines used in our review is adapted from the 2011 Institute of Medicine (now the National Academies of Medicine), but broadened to include all health considerations, not solely those related to healthcare and patient care: ‘statements that include recommendations, intended to optimize patient care, that are informed by a systematic review of evidence and an assessment of the benefits and harms of alternative care options’ [29].

### 3.3 Context

We included any human health guidelines and HTAs covering a wide range of clinical, health system, and public health topics that also address outcomes measuring impacts on planetary boundaries. We did not restrict eligibility based on geography or the level of government or region to which a guideline was targeted.

### 3.4 Types of Sources

This scoping review focused on health guidelines and HTAs that addressed planetary health outcomes including those that focused on animal health, natural systems, and the environment. We included the most recent version of each report when multiple versions were available. Methodological papers or handbooks offering insights into incorporating planetary health considerations into the guideline development or HTA process were also included. Moreover, health guidelines and HTAs published in any language were considered, with translation tools used to assess non‐English studies and reports.

### 3.5 Exclusion Criteria

We excluded studies or reports not directly related to planetary health, including those unrelated to health guidelines or HTAs. Life cycle assessment (LCA) modelling studies that were not part of a guideline or HTA were also excluded. Abstracts that did not explicitly reference planetary health, one health, ecosystem health, climate change, environmental sustainability or related concepts were excluded during title and abstract screening. Additionally, concepts related to sustainability that were not relevant to environmental or planetary health, such as sustainable financing, were excluded. Lastly, studies focusing solely on the impact of climate change or the environment on health were not included in our review.

### 3.6 Literature Search

The search strategy was developed in consultation with a health sciences librarian at McMaster University. All database searches were conducted from inception to September 2023. The search identified health guidelines and HTAs that have addressed planetary health outcomes or considerations. We completed a primary search of the literature using the following databases: Ovid MEDLINE, EMBASE, CINAHL, Global Health, Health Systems Evidence, Greenfile and Environmental Issues. We complemented this search with the GIN international guideline library and registry of guidelines in development [30], BIGG international database of GRADE guidelines [31], Epistemonikos GRADE guidelines repository [32], GRADEpro GDT Database of GRADE EtD's and Guidelines [33], MAGICapp [34], National Institute for Health and Care Excellence (NICE) website [35] and the World Health Organization website [36]. We also conducted a search for online content on Google using incognito mode with similar search terms to identify any unpublished documents of relevance. In addition, the search was further expanded by utilizing a previous scoping review of handbooks on guideline development to search for relevant references to planetary health. Planetary health terms included ‘planetary health’ OR ‘planet* health’ OR ‘ecosystem’ OR ‘one health’ OR ‘climate change*’ OR ‘health’ OR ‘environment* health’ OR ‘sustainab*’ OR ‘carbon footprint’. Guideline terms included ‘practice guideline’ OR ‘guideline*’ OR ‘consensus*’ OR ‘health plan* guideline’ OR ‘position statement?’ among others. HTA terms included ‘biomedical technology assessment’ OR ‘health tech* assessment’ OR ‘health* technology assessment’ among others. Full search strategy for the different databases has been provided in the Appendix S2.

### 3.7 Evidence Selection

A web‐based platform, Covidence [37], was used to remove duplicates and screen the retrieved articles. Screening was piloted in meetings with five to 10 reviewers. Title, abstract, and full‐text screening were done by two independent reviewers in duplicate. Disagreements were resolved through discussion among reviewers or with a senior reviewer. A simple screening algorithm was employed. The reasons for exclusion were documented in the full‐text screening phase.

### 3.8 Data Extraction

A pilot‐tested and standardized form was used to extract data from included guidelines. One reviewer performed preliminary data extraction, the results of which were then verified by a second reviewer (M.R. and T.P.). Any disagreements were resolved through discussion or involving a third reviewer. The primary investigator of the project reviewed and refined the extracted data for each guideline. Data were gathered on study characteristics, planetary health considerations, intervention details, and funding and conflict of interest disclosures. A detailed list can be found in the published protocol [24].

### 3.9 Data Analysis and Presentation

We conducted a descriptive and thematic synthesis of the four included guidelines. Rather than applying a formal qualitative coding framework, our analysis was driven by iterative team discussions among four members (A.J.D., F.A.M., M.R., and T.P.) over three meetings. Each member independently reviewed the full texts and recorded observations using an extraction template. Notes and insights from each reviewer were synthesized to identify broad themes relevant to planetary health considerations, including areas such as goods production and transport, patient/staff travel, diagnostic testing, facility requirements, IPAC measures and disposal impacts. To promote consistency and transparency, we defined and refined themes during team discussions, ensuring they were consistently applied across the included guidelines. Any discrepancies in interpretation were resolved through consensus. The themes were not developed through line‐by‐line coding, but emerged through collaborative review, note‐taking and group reflection.

We also used descriptive statistics, such as frequencies and percentages, to summarize the data. Results of the analysis were presented narratively, with classifications for each topic presented in a table, as appropriate, and the quantified data presented in graphs.

The risk of bias and the quality of the studies were assessed for health guidelines/HTAs using the Appraisal of Guidelines for Research and Evaluation II (AGREE II) instrument by two trained independent reviewers [38]. Any discrepancies were resolved through consensus between reviewers. Consensus judgments are reported for each item by guideline. The quality of the theoretical papers or commentaries included in the scoping review was not assessed, as it is not typically done in scoping reviews [20, 39].

## 4 Results

Out of 26,291 studies identified, we included 38 studies meeting criteria (Appendix S3). Summarized characteristics of included studies are shown in Table 1. A list of included studies can be found in the Appendix S4. The most common study type was commentary (*N* = 14, 37%). Six studies (16%) were a planetary health impact evaluation of a guideline or HTA and another six (16%) were methodological papers addressing planetary health. Four (11%) were health guidelines and the remainder provided input on methods, evaluation or comment on the consideration of planetary health in health guidelines or HTAs.

**Table 1:** Summarized characteristics of included studies.

	Number (%) (*N* = 38)
**Study type**	
Commentary	14 (37%)
Planetary Health Impact Evaluation of Guideline/HTA	6 (16%)
Methodological Paper	6 (16%)
Guideline	4 (11%)
Narrative Review	4 (11%)
Scoping Review	2 (5%)
Guideline Implementation Evaluation	1 (3%)
Qualitative Study	1 (3%)
**Topic/discipline**	
Nutrition	15 (39%)
HTA Methods	14 (37%)
Anesthesia	2 (5%)
Dermatology	2 (5%)
Economics	1 (3%)
Gastroenterology	1 (3%)
Guideline Methods	1 (3%)
Healthcare Sustainability Certification	1 (3%)
Respirology	1 (3%)
**Country/region of Production**	
UK	8 (21%)
Australia	7 (18%)
Spain	5 (13%)
Germany	4 (11%)
Canada	2 (5%)
China	2 (5%)
Multiple	2 (5%)
The Netherlands	1 (3%)
Brazil	1 (3%)
Finland	1 (3%)
France	1 (3%)
Qatar	1 (3%)
Sweden	1 (3%)
Switzerland	1 (3%)
United States	1 (3%)

### 4.1 Characteristics of Included Studies

**Figure 1:**
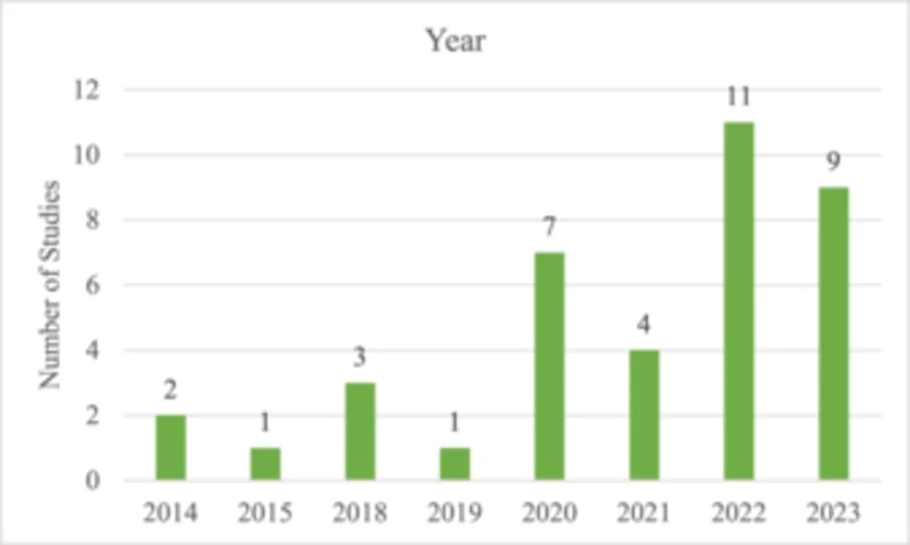
Year of publication of included studies (*N* = 38).

Of the 38 included studies, 15 (40%) addressed nutrition‐related topics. The largest number of included studies (*N* = 8, 21%) had a topic focus on or lead author originating in the United Kingdom, with a close second coming from Australia (*N* = 7, 18%). Only one study, from Brazil, did not originate from or focus on a high‐income country setting [40]. More than half of the studies (*N *= 21, 53%) were published between 2022 and 2023 (Figure 1). Commentaries primarily focused on the importance of incorporating planetary health considerations into health guidelines and HTAs, or challenges of doing so. The health guidelines [41, 42, 43, 44] had incorporated planetary health considerations while formulating recommendations but no such HTAs were found. Included methodological studies primarily focused on dimensions of planetary health considerations to incorporate into HTAs, with one methodological study [10] providing guidance for health guidelines.

Table 2 presents the planetary outcome categories and measures. The majority (*N* = 29, 76%) of included studies addressed CO_2_ emissions as a planetary health outcome measure, while 15 (39%) studies addressed other greenhouse gas emissions. Twelve (32%) studies considered water use and 10 (26%) studies considered waste production. The majority of the studies directly considered environmental health outcomes (*N* = 30, 79%) and human health outcomes (*N *= 23, 61%), but few addressed animal/one health outcomes (*N* = 4, 11%) and natural systems (*N* = 3, 8%). Table 3 demonstrates the number of included studies that address outcomes across the planetary boundary dimensions and stages of the life cycle. Most studies addressed climate change‐oriented planetary boundaries, while some addressed biosphere integrity and freshwater. Very few studies addressed all stages in a healthcare life cycle [42, 45] with most focusing only on goods production, transport, and disposal.

**Table 2:** Identified outcomes considered by included studies.

Planetary health outcome	Number (%) (*N* = 38)
**Categories**	
Environmental Health	30 (79%)
Human Health	23 (61%)
Animal/One Health	4 (11%)
Natural Systems	3 (8%)
**Measures**	
CO_2_ Emissions	29 (76%)
Other Greenhouse Gas Emissions	15 (39%)
Water Use	12 (32%)
Waste Production	10 (26%)
Methane Emissions	5 (13%)
Energy Input	5 (13%)
Nitrogen/Phosphorous Inputs	4 (11%)
Monetary Equivalents of Impacts	3 (8%)

**Table 3:** Planetary boundary and life cycle stage of outcomes identified by the number of included studies.^a^

Health life cycle\planetary boundary	Climate change	Biosphere integrity	Stratospheric ozone depletion	Ocean acidification	Biogeochemical flows	Land‐system change	Freshwater use	Atmospheric aerosol loading	Novel entities
Goods production	14	9	3	0	1	4	8	2	0
Goods transport	11	6	3	0	0	1	7	2	0
Patient/staff travel	3	1	0	0	0	0	0	0	0
Diagnostic tests	3	1	0	0	0	0	0	0	0
Facility requirements	2	1	0	0	0	0	0	0	0
IPAC requirements	2	1	0	0	0	0	0	0	0
Disposal impacts	13	8	2	0	1	4	7	2	0

^a^Shade of green colour conditionally formatted to demonstrate larger numbers as heat map.

### 4.2 Description and Quality of Guidelines

Table 4 demonstrates the characteristics of the four included guidelines. The median AGREE II score was 52%, with one study that had the highest AGREE II score partially using GRADE methods to assess the certainty of evidence [43]. See Appendix S5 for AGREE‐II scores by domain.

**Table 4:** Characteristics of included guidelines.

Guideline	Use of GRADE approach?	Description of study approach	AGREE II Score (%)
Aas 2023 [43]	Partial	Diabetes diet guidelines with GRADE certainty ratings, though without utilizing GRADE EtD frameworks. Recommendations incorporate considerations for minimal environmental impact, promoting sustainable dietary choices while informing diabetes management. Formulation of PICO questions unclear.	108 (67%)
Rodriguez DeSantiago 2022 [42]	No	Systematic literature review and expert consensus (Delphi method) used to assess the environmental impact of gastrointestinal endoscopy. Structured voting process conducted, with statements requiring at least 80% agreement for inclusion.	99 (61%)
White 2022 [44]	No	Modified Delphi process to ensure broad expert agreement on environmentally sustainable anesthesia. 634 proposed principles condensed into 7 core principles on environmentally sustainable practices.	68 (42%)
Wu 2022 [41]	No	Used life cycle assessment to evaluate environmental impacts. Compared changes in 5 environmental impact and nutritional quality indicators.	26 (16%)
Median AGREE‐II score			83·5 (52%)

### 4.3 Narrative Summary

Below, we present the results of the scoping review as a narrative synthesis organized around three key findings from health guidelines and HTAs: (1) the normative significance of addressing planetary health; (2) the scope, quality, and methods of included guidelines and HTAs; (3) inconsistencies in development methods and (4) other challenges in addressing planetary health.

#### 4.3.1 The Normative Significance of Addressing Planetary Health in Health Guidelines and HTAs

More than half of the identified studies were published between 2022 and 2023, suggesting a recent increase in focus on planetary health in health guidelines. Several studies emphasize the importance of planetary health at normative and standard‐setting levels. For example, Rose et al highlight in the Position Paper for the Society for Nutrition Education and Behaviour that ‘federal dietary guidelines should include environmental sustainability considerations’ [46]. Other included studies also emphasize the normative importance of addressing environmental impact, such as the statement: ‘Anaesthesia practice makes a measurable contribution to global warming […] To protect public health, anaesthesia providers need to reduce the contribution their practice makes to global warming’ [44].

#### 4.3.2 Scope, Quality, and Methods of Included Health Guidelines and HTAs

Of the 38 included studies, we identified four health guidelines (10·5%) considering planetary health [41, 42, 43, 44] and 34 studies (89·5%) that discussed or provided advice on incorporating the concept of planetary health into health guidelines or HTAs, but did not present its application. They varied in their methodological approach and did not consistently provide actionable statements on planetary health. Moreover, none of the included studies discussed assessing the certainty or quality of planetary health evidence.

Twenty‐nine of the 38 (76·3%) studies focused primarily on carbon‐based outcomes, such as the CO_2_ emissions. For these studies, while broad planetary health impacts were often mentioned, a rationale for exclusion of other planetary health boundary‐oriented outcomes was not provided. Eight studies (21·1%) addressed freshwater use, primarily within the context of LCAs. These studies mainly focused on water inputs, rather than the impact of outputs on freshwater resources.

#### 4.3.3 Inconsistencies in Methods for Developing Health Guidelines and HTAs

Of the four guidelines addressing planetary health, only one used GRADE to assess the certainty in the evidence [43]. Another guideline referenced that their specialty society typically develops guidelines using the GRADE approach. However, they did not consider this approach for planetary health, as they stated that it was not appropriate given the lack of underlying evidence for the planetary health recommendations they were developing [42]. None of the guidelines included specific planetary health or related discipline experts.

One methodological approach for assessing planetary health discussed in an HTA involved integrating carbon footprints and the social cost of carbon (i.e., cost to society of carbon emissions) into HTA economic evaluations or budget impact assessments. For example, “The UK NHS has reported that propellants from inhalers account for 8% of the NHS's entire carbon footprint. More than 640 million inhalers are used globally every year. However, product HTA and appraisals typically favour low‐cost inhalers and do not consider benefits to the system, such as less waste and less CO_2_e. Budget Impact Analyses may include the monetary value of a more favourable PCF using a social cost of carbon (SCC), estimated at 36 to 220 US$/tCO_2_e. Such analysis may inform decisions which contribute to the national efforts to reduce CO_2_ emissions.” [47]

One included study reported that environmental impacts are not a standard criteria considered by HTAs, but could be incorporated in ‘other criteria’ that are sometimes considered [48]. It also described proposals for incorporating planetary health considerations into HTAs, such as using flexible or dynamic willingness‐to‐pay thresholds for products that could improve healthcare sustainability [48].

#### 4.3.4 Other Challenges in Addressing Planetary Health

Several studies noted the limited availability of data for assessing planetary health outcomes [41, 46]. Most studies focused on GHG emissions, while data for other planetary health outcomes, such as biodiversity loss, land use, or chemical pollution, were less frequently reported. Most studies addressed short‐term outcomes including health outcomes that generally have a shorter follow‐up period while planetary health outcomes unfold over decades (Table 2) [41]. Some studies reported a lack of practical guidance for implementing planetary health recommendations within health guidelines [46]. Reported barriers to implementation included the need for behavioural changes, such as dietary modifications [46].

## 5 Discussion

### 5.1 Main Findings

Our scoping review identified 38 studies on the topic of planetary health, including four clinical guidelines that explicitly considered planetary health in their recommendations. Most studies addressed climate‐change‐related planetary boundaries with GHG emissions being the most common planetary health outcome. A key limitation in the reviewed guidelines was the narrow scope of planetary health boundaries considered. While four of the six transgressed boundaries (climate change, biosphere integrity, freshwater use, and land‐system change) [2] were mentioned in several studies, other transgressed boundaries—such as biogeochemical flows or introduction of novel entities—were rarely considered. This omission may be due to the absence of relevant planetary health experts in guideline panels, overlooking other key planetary boundaries. Moreover, the approaches used to integrate planetary health varied across guidelines, with no standardized methodology in place. Of the four guidelines, only one applied the GRADE methodology to assess evidence and develop recommendations.

### 5.2 Strengths and Limitations

We are aware of one other published scoping review that looked at the incorporation of environmental and sustainability considerations in guidelines and HTAs as part of a broader effort by the National Institute for Health and Care Excellence (NICE) in the United Kingdom [49]. However, to our knowledge, our study is the first to take a broader planetary health perspective, systematically assessing how various planetary boundaries are integrated into clinical and public health guidelines and HTAs, and identifying key gaps in expert engagement and methodological standardization. We conducted a rigorous scoping review using inclusive search terms and screening criteria to map out the landscape of guidelines that have considered planetary health. We also utilized analytical frameworks to characterize included studies in terms of their consideration of all planetary boundaries and impacts of interventions across a full life cycle.

Our review has several limitations as well. First, we only found four health guidelines and no HTAs which may limit the generalizability of our review. Second, we searched for related terms, such as sustainability, climate change, and environmental health, but due to conceptual challenges in defining an emerging term like planetary health, we may have missed some studies. Third, we may not have captured some relevant guidelines and HTAs because we did not formally search grey literature, where such documents may be published rather than in peer‐reviewed journals. Future research will expand the use of non‐peer‐reviewed databases and internet searches to strengthen the inclusion of guidelines and HTAs. This may also reflect publication bias, as guidelines addressing planetary health are less likely to appear in academic literature. However, we tried to identify relevant studies through expert engagement. Lastly, the coding of our study topics and consideration of planetary health was limited by the reported information included in manuscripts, that is, if a manuscript did not explicitly address the topic, we were unable to classify it.

### 5.3 Implications for Policy, Practice, and Research

This review highlighted several key gaps in the incorporation of planetary health into health guidelines and potentially HTAs.

First, without validated tools, the consideration of planetary health in health guidelines and HTAs remains inconsistent, leading to variable recommendations hindering effective policy implementation. Researchers should prioritize developing standardized frameworks to enhance consistency, transparency, and comparability across studies. Since the GRADE framework plays a central role in systematizing guideline development, integrating planetary health into its EtD framework would promote standardization and ensure that environmental impacts are systematically evaluated where needed.

Second, further studies should explore the specific expertise and skillset required to address planetary health within guidelines and HTAs, identifying gaps in current knowledge and developing strategies to engage environment and sustainability experts in these processes. Third, researchers should assess the trade‐offs between clinical and planetary health outcomes to better understand how the various interest‐holders prioritize these factors. Without it, the weighing of different human and planetary health outcomes could be challenging if, for instance, an intervention improves human health but worsens planetary health. Lastly, there is a need to quantify resource inputs to determine feasibility and acceptability of integrating planetary health into health guidelines and HTAs. Given the increasing interest in planetary health, an update of this study will be necessary to assess evolving research and methodological developments in health guidelines and HTAs.

Despite the growing interest, however, we note that there have been limited applications of this integration in practice. Only four guidelines in our review explicitly incorporated planetary health, while the remainder studies provided insights and discussions surrounding this topic. Even when planetary health considerations are included, guidelines often lack implementation strategies making it difficult for health professionals and policymakers to enact recommendations.

Some planetary health impacts, such as CO_2_ emissions, are both local and global, while others, such as freshwater use or pollution, maybe more local. Policymakers must consider how to balance national and international priorities when integrating planetary health into healthcare decision‐making without exacerbating global inequalities.

There is an urgent need for clear methodological guidance on incorporating planetary health into health guidelines and HTAs, as well as a need for the development of quality appraisal tools to assess planetary health methods. Our GRADE for Planetary Health Project Group aims to address this knowledge gap. Such tools would improve clarity in development and reporting, while enhancing accountability and comparability across studies. This would also require the training of researchers and guideline developers to understand where planetary health methods are applicable, selecting appropriate methods for evaluation, and accounting for contextual factors relevant to different guidelines and HTAs. Government and health agencies could also consider LCAs during drug regulation while incentivizing environmentally sustainable practices to promote the uptake of planetary health components into healthcare decision‐making.

## 6 Conclusion

Our findings highlight that while planetary health dimensions and outcomes, particularly CO_2_ emissions, are frequently addressed, others such as biodiversity loss, land‐system changes, and chemical pollution are largely overlooked. Methodological inconsistencies persist, with no standardized approach for integrating or assessing planetary health into guidelines or HTAs.

As interest in planetary health continues to grow, future work should focus on developing methodological frameworks for incorporating planetary health, improving reporting standards, and incorporating interdisciplinary expertise to ensure planetary health is systematically embedded in healthcare decision‐making.

## Author Contributions

P.S., M.R., and T.P. made substantial contributions to the study conceptualization, design, data collection, analysis, and decision to publish. G.I.L., I.N., H.S., F.A.M., and H.J.S. made contributions to the study design. C.T.J.M., A.H., K.A.S., A.J.D., L.M.J., K.M.S., H.N., R.H., and R.L.M. made contributions to the data collection and analysis. P.S., M.R., A.J.D., and T.P. contributed to writing the original draft. All authors gave feedback on the revised report and approved the final version of the manuscript. T.P. is the guarantor of this study.

## Ethics Statement

The authors have nothing to report.

## Conflicts of Interest

Alina Herrmann is a member of the German Alliance of Climate Change and Health (KLUG), and honorary spokesperson on climate change and health at the German Society of General and Family Medicine (DEGAM). Hendrik Napierala is a member of the Planetary Health Working Group of the Association of Scientific Medical Societies in Germany (AWMF) and a member of the climate change and health section at the German Society of General Practice and Family Medicine (DEGAM). Holger Schünemann is the GRADE working group cochair. Ruben Heuer is a member of the Planetary Health Working Group of the Association of Scientific Medical Societies in Germany (AWMF). All other authors declare no conflicts of interest.

## Supporting information


**Supplementary Appendix S1:** Preferred Reporting Items for Systematic reviews and Meta‐Analyses extension for Scoping Reviews (PRISMA‐ScR) Checklist. **Supplementary Appendix S2:** Search Strategy. **Supplementary Appendix S3:** PRISMA flow chart. **Supplementary Appendix S4:** List of included studies. **Supplementary Appendix S5:** AGREE‐II scores by domain.

## Data Availability

The datasets used and/or analysed during the current study are available from the corresponding author upon a reasonable request.

## References

[1] A. J. MacNeill , F. McGain , and J. D. Sherman , “Planetary Health Care: A Framework for Sustainable Health Systems,” Lancet Planetary Health 5, no. 2 (2021): e66–e68. 33581064 10.1016/S2542-5196(21)00005-X

[2] K. Richardson , W. Steffen , W. Lucht , et al., “Earth Beyond Six of Nine Planetary Boundaries,” Science Advances 9, no. 37 (2023): eadh2458. 37703365 10.1126/sciadv.adh2458 PMC10499318

[3] M. Romanello , C. Napoli , C. Green , et al., “The 2023 Report of the Lancet Countdown on Health and Climate Change: The Imperative for a Health‐Centred Response in a World Facing Irreversible Harms,” Lancet 402, no. 10419 (2023): 2346–2394. 37977174 10.1016/S0140-6736(23)01859-7 PMC7616810

[4] D. Duong , “How Canadian Hospitals Are Decreasing Carbon Emissions,” Canadian Medical Association Journal 195, no. 16 (2023): E594. 37094870 10.1503/cmaj.1096048 PMC10125190

[5] M. J. Eckelman and J. Sherman , “Environmental Impacts of the U.S. Health Care System and Effects on Public Health,” PLOS ONE 11, no. 6 (June 2016): e0157014. 27280706 10.1371/journal.pone.0157014 PMC4900601

[6] M. Lenzen , A. Malik , M. Li , et al., “The Environmental Footprint of Health Care: A Global Assessment,” Lancet Planetary Health 4, no. 7 (2020): e271–e279. 32681898 10.1016/S2542-5196(20)30121-2

[7] J. Ranganathan , L. Corbier , P. Bhatia , et al., The Greenhouse Gas Protocol: A Corporate Accounting and Reporting Standard , Revised Edition. (World Business Council on Sustainable Development (WBCSD) and World Resources Institute (WRI), 2004). https://ghgprotocol.org/corporate‐standard.

[8] V. Rossa‐Roccor , E. S. Acheson , F. Andrade‐Rivas , et al., “Scoping Review and Bibliometric Analysis of the Term ‘Planetary Health’ in the Peer‐Reviewed Literature,” Front Public Health 8 (2020): 343, 32850584 10.3389/fpubh.2020.00343 PMC7403469

[9] S. Whitmee , A. Haines , C. Beyrer , et al., “Safeguarding Human Health in the Anthropocene Epoch: Report of The Rockefeller Foundation–Lancet Commission on Planetary Health,” Lancet 386, no. 10007 (2015): 1973–2028. 26188744 10.1016/S0140-6736(15)60901-1

[10] A. Herrmann , B. Lenzer , B. S. Müller , et al., “Integrating Planetary Health Into Clinical Guidelines to Sustainably Transform Health Care,” Lancet Planetary Health 6, no. 3 (2022): e184–e185. 35278382 10.1016/S2542-5196(22)00041-9

[11] I. Neumann and H. Schünemann , The GRADE Book , Vol. 1. (The GRADE Working Group), [Cited 2025 Feb 9], https://book.gradepro.org.

[12] R. L. Morgan , L. Kelley , G. H. Guyatt , A. Johnson , and J. N. Lavis , “Decision‐Making Frameworks and Considerations for Informing Coverage Decisions for Healthcare Interventions: A Critical Interpretive Synthesis,” Journal of Clinical Epidemiology 94 (2018): 143–150. 28988959 10.1016/j.jclinepi.2017.09.023

[13] P. Alonso‐Coello , H. J. Schünemann , J. Moberg , et al., “GRADE Evidence to Decision (EtD) Frameworks: A Systematic and Transparent Approach to Making Well Informed Healthcare Choices. 1: Introduction,” BMJ 353 (2016): i2016. 27353417 10.1136/bmj.i2016

[14] T. Piggott , G. I. Leontiadis , A. Herrmann , et al., “We'Re Living Through a Planetary Health Crisis: Health Guidelines Must Consider Planetary Health,” Lancet Planetary Health 8, no. 12 (2024): e979–e980. 39674202 10.1016/S2542-5196(24)00300-0

[15] V. G. Le , M. K. Nguyen , C. Lin , et al., “Review on Personal Protective Equipment: Emerging Concerns in Micro(Nano)Plastic Pollution and Strategies for Addressing Environmental Challenges,” Environmental Research 257 (2024): 119345. 38851370 10.1016/j.envres.2024.119345

[16] E. Andrews , D. Pearson , C. Kelly , L. Stroud , and M. Rivas Perez , “Carbon Footprint of Patient Journeys Through Primary Care: A Mixed Methods Approach,” British Journal of General Practice 63, no. 614 (2013): e595–e603. 10.3399/bjgp13X671579 PMC3750798 23998839

[17] L. Wang , S. Li , I. M. Ahmad , et al., “Global Face Mask Pollution: Threats to the Environment and Wildlife, and Potential Solutions,” Science of the Total Environment 887 (2023): 164055. 37178835 10.1016/j.scitotenv.2023.164055 PMC10174332

[18] H. J. Schünemann , S. Brennan , E. A. Akl , et al., “The Development Methods of Official GRADE Articles and Requirements for Claiming the Use of GRADE—A Statement by the GRADE Guidance Group,” Journal of Clinical Epidemiology 159 (2023): 79–84. 37211327 10.1016/j.jclinepi.2023.05.010

[19] M. D. J. Peters , C. Marnie , A. C. Tricco , et al., “Updated Methodological Guidance for the Conduct of Scoping Reviews,” JBI Evidence Synthesis 18, no. 10 (2020): 2119–2126. 33038124 10.11124/JBIES-20-00167

[20] M. D. J. Peters , C. Godfrey , P. McInerney , et al., “Best Practice Guidance and Reporting Items for the Development of Scoping Review Protocols,” JBI Evidence Synthesis 20, no. 4 (2022): 953–968. 35102103 10.11124/JBIES-21-00242

[21] A. C. Tricco , E. Lillie , W. Zarin , et al., “PRISMA Extension for Scoping Reviews (PRISMA‐ScR): Checklist and Explanation,” Annals of Internal Medicine 169, no. 7 (2018): 467–473. 30178033 10.7326/M18-0850

[22] M. J. Page , J. E. McKenzie , P. M. Bossuyt , et al., “The PRISMA 2020 Statement: An Updated Guideline for Reporting Systematic Reviews,” BMJ 372 (2021): n71. 33782057 10.1136/bmj.n71 PMC8005924

[23] M. Peters , C. Godfrey , P. McInerney , Z. Munn , A. Tricco , and H. Khalil . “Scoping Reviews,” in *JBI Manual for Evidence Synthesis*, (2020), [Cited 2025 Feb 9], https://synthesismanual.jbi.global. 10.46658/JBIMES-24-01. 33038124

[24] T. Piggott , M. Raja , C. T. J. Michels , et al., “Considering Planetary Health in Health Guidelines and Health Technology Assessments: A Scoping Review Protocol,” Systematic Reviews 13, no. 1 (2024): 163. 38909251 10.1186/s13643-024-02577-2 PMC11193899

[25] B. Talukder , N. Ganguli , E. Choi , M. Tofighi , G. W. vanloon , and J. Orbinski , “Exploring the Nexus: Comparing and Aligning Planetary Health, One Health, and EcoHealth,” Global Transitions 6 (2024): 66–75.

[26] L. Caesar , B. Sakschewski , L. Andersen , et al., Planetary Health Check: A Scientific Assessment of the State of the Planet [Internet] (Potsdam Institute for Climate Impact Research, 2024), 1–93, https://www.planetaryhealthcheck.org/storyblok‐cdn/f/301438/x/a4efc3f6d5/planetaryhealthcheck2024_report.pdf.

[27] W. Steffen , K. Richardson , J. Rockström , et al., “Planetary Boundaries: Guiding Human Development on a Changing Planet,” Science 347, no. 6223 (2015): 1259855. 25592418 10.1126/science.1259855

[28] J. Rockström , W. Steffen , K. Noone , et al., “Planetary Boundaries: Exploring the Safe Operating Space for Humanity,” Ecology & Society 14, no. 2 (2009): 32, https://www.jstor.org/stable/26268316.

[29] R. Graham , M. Mancher , D. Wolman , S. Greenfield , and E. Steinberg , Clinical Practice Guidelines We can Trust (The National Academies Press, 2011). 24983061

[30] Guidelines International Network (GIN) [Internet], https://guidelines.ebmportal.com/.

[31] BIGG International Database of GRADE Guidelines [Internet]. [Cited 2025 Feb 12], https://sites.bvsalud.org/bigg/en/biblio/. 10.1016/j.lana.2021.100099 PMC8863112 35233554

[32] Epistemonikos: Database of the Best Evidence‐Based Health Care [Internet]. GRADE Guidelines Repository. [Cited 2025 Feb 12], https://www.epistemonikos.org/en/groups/grade_guideline.

[33] GRADEpro GDT Database of GRADE EtD's and Guidelines [Internet]. Database of GRADE EtD's and Guidelines. [Cited 2025 Feb 12], https://guidelines.gradepro.org/search.

[34] MAGICapp [Internet]. A Digital Authoring and Publication Platform for the Evidence Ecosystem, by MAGIC Evidence Ecosystem Foundation. [Cited 2025 Feb 12], https://app.magicapp.org/#/guidelines.

[35] National Institute for Health and Care Excellence (NICE) [Internet] . Published: Guidance, Quality Standards and Advice (NICE, 2021), https://www.nice.org.uk/guidance/published.

[36] Publications [Internet]. World Health Organization. [Cited 2025 Feb 12], https://www.who.int/publications/i.

[37] Covidence [Internet]. Covidence. [Cited 2025 Feb 12], https://www.covidence.org/.

[38] M. C. Brouwers , M. E. Kho , G. P. Browman , et al., “AGREE II: Advancing Guideline Development, Reporting and Evaluation in Health Care,” Canadian Medical Association Journal 182, no. 18 (2010): E839–E842. 20603348 10.1503/cmaj.090449 PMC3001530

[39] D. Pollock , E. L. Davies , M. D. J. Peters , et al., “Undertaking a Scoping Review: A Practical Guide for Nursing and Midwifery Students, Clinicians, Researchers, and Academics,” Journal of Advanced Nursing 77, no. 4 (2021): 2102–2113. 33543511 10.1111/jan.14743 PMC8049063

[40] World Bank Country and Lending Groups [Internet]. The World Bank. [Cited 2025 Feb 13], https://datahelpdesk.worldbank.org/knowledgebase/articles/906519‐world‐bank‐country‐and‐lending‐groups.

[41] H. Wu , G. K. MacDonald , J. N. Galloway , et al., “A new Dietary Guideline Balancing Sustainability and Nutrition for China's Rural and Urban Residents,” iScience 25, no. 10 (2022): 105048. 36185362 10.1016/j.isci.2022.105048 PMC9519510

[42] E. Rodríguez de Santiago , M. Dinis‐Ribeiro , H. Pohl , et al., “Reducing the Environmental Footprint of Gastrointestinal Endoscopy: European Society of Gastrointestinal Endoscopy (ESGE) and European Society of Gastroenterology and Endoscopy Nurses and Associates (ESGENA) Position Statement,” Endoscopy 54, no. 8 (2022): 797–826. 35803275 10.1055/a-1859-3726

[43] A. M. Aas , M. Axelsen , C. Churuangsuk , et al., “Evidence‐Based European Recommendations for the Dietary Management of Diabetes,” Diabetologia 66, no. 6 (2023): 965–985. 37069434 10.1007/s00125-023-05894-8

[44] S. M. White , C. L. Shelton , A. W. Gelb , et al., “Principles of Environmentally‐Sustainable Anaesthesia: A Global Consensus Statement From the World Federation of Societies of Anaesthesiologists,” Anaesthesia 77, no. 2 (2022): 201–212. 34724710 10.1111/anae.15598 PMC9298028

[45] B. A. K. Pekarsky , “The Inclusion of Comparative Environmental Impact in Health Technology Assessment: Practical Barriers and Unintended Consequences,” Applied Health Economics and Health Policy 18, no. 5 (2020): 597–599. 32377983 10.1007/s40258-020-00578-5

[46] D. Rose , M. C. Heller , and C. A. Roberto , “Position of the Society for Nutrition Education and Behavior: The Importance of Including Environmental Sustainability in Dietary Guidance,” Journal of Nutrition Education and Behavior 51, no. 1 (2019): 3–15. 30635107 10.1016/j.jneb.2018.07.006 PMC6326035

[47] C. Miltenburger , F. Borgstrom , and O. Eklund , “Ecological Benefits of Health Technologies—How to Consider Public Value in Funding Decisions?,” Value in Health 21 (2018): S25.

[48] L. Ramjee , G. Tremblay , A. Forsythe , and K. Sandman , “PNS6 Saving Lives or the Environment ‐ How About Both? The Role of Health Technology Assessment (HTA) Bodies in Creating Sustainable Healthcare Systems,” supplement, Value in Health 24, no. Suppl 1 (2021): S173–S174.

[49] A. C. Pinho‐Gomes , S. H. Yoo , A. Allen , H. Maiden , K. Shah , and M. Toolan , “Incorporating Environmental and Sustainability Considerations Into Health Technology Assessment and Clinical and Public Health Guidelines: A Scoping Review,” International Journal of Technology Assessment in Health Care 38, no. 1 (2022): e84. 36510398 10.1017/S0266462322003282

