# Supplementary material for: Planetary Health in Health Guidelines and Health Technology Assessments: A Scoping Review
Source: Clin Public Health Guidel. 2025 Dec 13;3(1):e70056. doi: 10.1002/gin2.70056 (PMC13563324; doi:10.1002/gin2.70056)
Supplement: Supplementary file 1 [file GIN2-3-e70056-s001.docx]

**Supplementary Appendix**

**Planetary Health in Health Guidelines and Health Technology Assessments: A Scoping Review**

Pakeezah Saadat, Andrea J. Darzi, Maheen Raja, Charlotte T.J. Michels, Alina Herrmann, Karolina Anna Scahill, Laura M. Jewell, KM Saif-Ur-Rahman, Hendrik Napierala, Ruben Heuer, Rebecca L. Morgan, Grigorios I. Leontiadis, Ignacio Neumann, Fiona A. Miller, Holger J. Schünemann, Thomas Piggott

**Corresponding Author**

Thomas Piggott

Assistant Professor (PT)

Health Research Methods, Evidence, and Impact

McMaster University

Hamilton, Canada

Email: [piggott@mcmaster.ca](mailto:piggott@mcmaster.ca)

Table *of* Contents

[Supplementary Appendix S1. Preferred Reporting Items for Systematic reviews and Meta-Analyses extension for Scoping Reviews (PRISMA-ScR) Checklist	3](#_Toc209516594)

[Supplementary Appendix S2. Search Strategy	6](#_Toc209516595)

[MEDLINE, EMBASE, and Global Health	6](#_Toc209516596)

[CINAHL	7](#_Toc209516597)

[Greenfile	8](#_Toc209516598)

[Epistemonikos	10](#_Toc209516599)

[Additional search terms:	10](#_Toc209516600)

[Supplementary Appendix S3. PRISMA flow chart	11](#_Toc209516601)

[Supplementary Appendix S4. List of included studies	12](#_Toc209516602)

[Supplementary Appendix S5. AGREE-II scores by domain	15](#_Toc209516603)

# **Supplementary Appendix S1. Preferred Reporting Items for Systematic reviews and Meta-Analyses extension for Scoping Reviews (PRISMA-ScR) Checklist**

| **SECTION** | **ITEM** | **PRISMA-ScR CHECKLIST ITEM** | **REPORTED ON PAGE #** |
| --- | --- | --- | --- |
| **TITLE** | | | |
| Title | 1 | Identify the report as a scoping review. | 1 |
| **ABSTRACT** | | | |
| Structured summary | 2 | Provide a structured summary that includes (as applicable): background, objectives, eligibility criteria, sources of evidence, charting methods, results, and conclusions that relate to the review questions and objectives. | 5 |
| **INTRODUCTION** | | | |
| Rationale | 3 | Describe the rationale for the review in the context of what is already known. Explain why the review questions/objectives lend themselves to a scoping review approach. | 7-8 |
| Objectives | 4 | Provide an explicit statement of the questions and objectives being addressed with reference to their key elements (e.g., population or participants, concepts, and context) or other relevant key elements used to conceptualize the review questions and/or objectives. | 9 |
| **METHODS** | | | |
| Protocol and registration | 5 | Indicate whether a review protocol exists; state if and where it can be accessed (e.g., a Web address); and if available, provide registration information, including the registration number. | 10 |
| Eligibility criteria | 6 | Specify characteristics of the sources of evidence used as eligibility criteria (e.g., years considered, language, and publication status), and provide a rationale. | 10 |
| Information sources* | 7 | Describe all information sources in the search (e.g., databases with dates of coverage and contact with authors to identify additional sources), as well as the date the most recent search was executed. | 12 |
| Search | 8 | Present the full electronic search strategy for at least 1 database, including any limits used, such that it could be repeated. | Appendix |
| Selection of sources of evidence† | 9 | State the process for selecting sources of evidence (i.e., screening and eligibility) included in the scoping review. | 13 |
| Data charting process‡ | 10 | Describe the methods of charting data from the included sources of evidence (e.g., calibrated forms or forms that have been tested by the team before their use, and whether data charting was done independently or in duplicate) and any processes for obtaining and confirming data from investigators. | 14 |
| Data items | 11 | List and define all variables for which data were sought and any assumptions and simplifications made. | 14 + protocol |
| Critical appraisal of individual sources of evidence§ | 12 | If done, provide a rationale for conducting a critical appraisal of included sources of evidence; describe the methods used and how this information was used in any data synthesis (if appropriate). | 14 |
| Synthesis of results | 13 | Describe the methods of handling and summarizing the data that were charted. | 14 |
| **RESULTS** | | | |
| Selection of sources of evidence | 14 | Give numbers of sources of evidence screened, assessed for eligibility, and included in the review, with reasons for exclusions at each stage, ideally using a flow diagram. | 15 |
| Characteristics of sources of evidence | 15 | For each source of evidence, present characteristics for which data were charted and provide the citations. | 15 |
| Critical appraisal within sources of evidence | 16 | If done, present data on critical appraisal of included sources of evidence (see item 12). | 17 |
| Results of individual sources of evidence | 17 | For each included source of evidence, present the relevant data that were charted that relate to the review questions and objectives. | 15-16 |
| Synthesis of results | 18 | Summarize and/or present the charting results as they relate to the review questions and objectives. | 17-19 |
| **DISCUSSION** | | | |
| Summary of evidence | 19 | Summarize the main results (including an overview of concepts, themes, and types of evidence available), link to the review questions and objectives, and consider the relevance to key groups. | 20 |
| Limitations | 20 | Discuss the limitations of the scoping review process. | 20-21 |
| Conclusions | 21 | Provide a general interpretation of the results with respect to the review questions and objectives, as well as potential implications and/or next steps. | 23 |
| **FUNDING** | | | |
| Funding | 22 | Describe sources of funding for the included sources of evidence, as well as sources of funding for the scoping review. Describe the role of the funders of the scoping review. | 4 |

JBI = Joanna Briggs Institute; PRISMA-ScR = Preferred Reporting Items for Systematic reviews and Meta-Analyses extension for Scoping Reviews.

* Where *sources of evidence* (see second footnote) are compiled from, such as bibliographic databases, social media platforms, and Web sites.

† A more inclusive/heterogeneous term used to account for the different types of evidence or data sources (e.g., quantitative and/or qualitative research, expert opinion, and policy documents) that may be eligible in a scoping review as opposed to only studies. This is not to be confused with *information sources* (see first footnote).

‡ The frameworks by Arksey and O’Malley (6) and Levac and colleagues (7) and the JBI guidance (4, 5) refer to the process of data extraction in a scoping review as data charting*.*

§ The process of systematically examining research evidence to assess its validity, results, and relevance before using it to inform a decision. This term is used for items 12 and 19 instead of "risk of bias" (which is more applicable to systematic reviews of interventions) to include and acknowledge the various sources of evidence that may be used in a scoping review (e.g., quantitative and/or qualitative research, expert opinion, and policy document).

*From:* Tricco AC, Lillie E, Zarin W, O'Brien KK, Colquhoun H, Levac D, et al. PRISMA Extension for Scoping Reviews (PRISMAScR): Checklist and Explanation. Ann Intern Med. 2018;169:467–473. [doi: 10.7326/M18-0850](http://annals.org/aim/fullarticle/2700389/prisma-extension-scoping-reviews-prisma-scr-checklist-explanation).

# **Supplementary Appendix S2. Search Strategy**

## **MEDLINE, EMBASE, and Global Health**

| Search Number | Query | Results |
| --- | --- | --- |
| #1 | exp planetary health/ | 474 |
| #2 | (planet* health or ecosystem* health or one health or climate change* health or environment* health or sustainab* or carbon footprint).ab,ti. | 388748 |
| #3 | 1 or 2 | 388877 |
| #4 | exp practice guideline/ | 746539 |
| #5 | (((((((guideline* or consensus* or health plan* guideline or position statement? or policy statement? or scientific statement? or recommendation? or clinical practice guideline or clinical guideline* or health) adj2 guideline*) or practice) adj2 guideline*) or clinical) adj2 guideline*) or practice pattern*).ab,ti. | 1334958 |
| #6 | 4 or 5 | 1734082 |
| #7 | exp biomedical technology assessment/ | 29427 |
| #8 | (health tech* assessment or health* technology assessment or healthy technology eval* or health* technology analysis or biomedical technology assess* or health econ* evaluation or HTA).ab,ti. | 24957 |
| #9 | 7 or 8 | 46809 |
| #10 | 6 or 9 | 1775488 |
| #11 | 3 and 10 | 17100 |

## **CINAHL**

| Search Number | Query | Results |
| --- | --- | --- |
| S1 | (MH "Ecosystem+") | (10,814) |
| S2 | TI (planet* health or ecosystem* health or one health or climate change* health or environment* health or sustainab* or carbon footprint) OR AB (planet* health or ecosystem* health or one health or climate change* health or environment* health or sustainab* or carbon footprint) | (37,837) |
| S3 | S1 or S2 | (48,366) |
| S4 | (MH "Practice Guidelines") | (85,739) |
| S5 | TI (guideline* or consensus* or health plan* guideline or position statement? or policy statement? or scientific statement? or recommendation? or clinical practice guideline or clinical guideline* or health N2 guideline* or practice N2 guideline* or clinical N2 guideline* or practice pattern*) OR AB (guideline* or consensus* or health plan* guideline or position statement? or policy statement? or scientific statement? or recommendation? or clinical practice guideline or clinical guideline* or health N2 guideline* or practice N2 guideline* or clinical N2 guideline* or practice pattern*) | (349,636) |
| S6 | S4 OR S5 | (389,684) |
| S7 | (MH "Health Impact Assessment") | (867) |
| S8 | TI (health tech* assessment or health* technology assessment or healthy technology eval* or health* technology analysis or biomedical technology assess* or health econ* evaluation or HTA) OR AB (health tech* assessment or health* technology assessment or healthy technology eval* or health* technology analysis or biomedical technology assess* or health econ* evaluation or HTA) | (4,458) |
| S9 | S7 OR S8 | (5,316) |
| S10 | S6 OR S9 | (393,949) |
| S11 | S3 AND S10 | (4,810) |

## **Greenfile**

| Search Number | Search Term | Results |
| --- | --- | --- |
| 1 | (TI ((planet* health OR ecosystem* health OR one health OR climate change* health OR environment* health OR sustainab* OR carbon footprint))) OR (AB ((planet* health OR ecosystem* health OR one health OR climate change* health OR environment* health OR sustainab* OR carbon footprint))) | 86,043 |
| 2 | (TI ((guideline* OR consensus* OR health plan* guideline OR position statement? OR policy statement? OR scientific statement? OR recommendation? OR clinical practice guideline OR clinical guideline* OR health N2 guideline* OR practice N2 guideline* OR clinical N2 guideline* OR practice pattern*))) OR (AB ((guideline* OR consensus* OR health plan* guideline OR position statement? OR policy statement? OR scientific statement? OR recommendation? OR clinical practice guideline OR clinical guideline* OR health N2 guideline* OR practice N2 guideline* OR clinical N2 guideline* OR practice pattern*))) | 29,006 |
| 3 | (TI ((health tech* assessment OR health* technology assessment OR healthy technology eval* OR health* technology analysis OR biomedical technology assess* OR health econ* evaluation OR HTA))) OR (AB ((health tech* assessment OR health* technology assessment OR healthy technology eval* OR health* technology analysis OR biomedical technology assess* OR health econ* evaluation OR HTA))) | 25 |
| 4 | ((TI ((health tech* assessment OR health* technology assessment OR healthy technology eval* OR health* technology analysis OR biomedical technology assess* OR health econ* evaluation OR HTA))) OR (AB ((health tech* assessment OR health* technology assessment OR healthy technology eval* OR health* technology analysis OR biomedical technology assess* OR health econ* evaluation OR HTA)))) OR ((TI ((guideline* OR consensus* OR health plan* guideline OR position statement? OR policy statement? OR scientific statement? OR recommendation? OR clinical practice guideline OR clinical guideline* OR health N2 guideline* OR practice N2 guideline* OR clinical N2 guideline* OR practice pattern*))) OR (AB ((guideline* OR consensus* OR health plan* guideline OR position statement? OR policy statement? OR scientific statement? OR recommendation? OR clinical practice guideline OR clinical guideline* OR health N2 guideline* OR practice N2 guideline* OR clinical N2 guideline* OR practice pattern*)))) | 29,031 |
| 5 | (((TI ((health tech* assessment OR health* technology assessment OR healthy technology eval* OR health* technology analysis OR biomedical technology assess* OR health econ* evaluation OR HTA))) OR (AB ((health tech* assessment OR health* technology assessment OR healthy technology eval* OR health* technology analysis OR biomedical technology assess* OR health econ* evaluation OR HTA)))) OR ((TI ((guideline* OR consensus* OR health plan* guideline OR position statement? OR policy statement? OR scientific statement? OR recommendation? OR clinical practice guideline OR clinical guideline* OR health N2 guideline* OR practice N2 guideline* OR clinical N2 guideline* OR practice pattern*))) OR (AB ((guideline* OR consensus* OR health plan* guideline OR position statement? OR policy statement? OR scientific statement? OR recommendation? OR clinical practice guideline OR clinical guideline* OR health N2 guideline* OR practice N2 guideline* OR clinical N2 guideline* OR practice pattern*))))) AND ((TI ((planet* health OR ecosystem* health OR one health OR climate change* health OR environment* health OR sustainab* OR carbon footprint))) OR (AB ((planet* health OR ecosystem* health OR one health OR climate change* health OR environment* health OR sustainab* OR carbon footprint)))) | 4,253 |

## **Epistemonikos**

| Search Number | Search Term |
| --- | --- |
| 1 | Title/abstract: (title:((((((((guideline* OR consensus* OR health plan* guideline OR position statement? OR policy statement? OR scientific statement? OR recommendation? OR clinical practice guideline OR clinical guideline* OR health) adj2 guideline*) OR practice) adj2 guideline*) OR clinical) adj2 guideline*) OR practice pattern*)) OR abstract:((((((((guideline* OR consensus* OR health plan* guideline OR position statement? OR policy statement? OR scientific statement? OR recommendation? OR clinical practice guideline OR clinical guideline* OR health) adj2 guideline*) OR practice) adj2 guideline*) OR clinical) adj2 guideline*) OR practice pattern*))) OR (title:((health tech* assessment OR health* technology assessment OR healthy technology eval* OR health* technology analysis OR biomedical technology assess* OR health econ* evaluation OR HTA)) OR abstract:((health tech* assessment OR health* technology assessment OR healthy technology eval* OR health* technology analysis OR biomedical technology assess* OR health econ* evaluation OR HTA))) |
| 2 | AND |
| 3 | Title/Abstract: (planet* health or ecosystem* health or one health or climate change* health or environment* health or sustainab* or carbon footprint) |

## Additional search terms

For GIN registry, BIGG international database, GRADEpro, MAGICApp, WHO website, Google Search (Incognito), where logic-based searches are not permitted; each separate search was completed: (planetary health OR climate change OR environmental health OR sustainability OR carbon footprint) + (guideline OR health technology assessment).

# **Supplementary Appendix S3. PRISMA flow chart**

**Identification of studies via databases and registers**

Records identified from:

Databases and Registers

(n = 26,291)

Records removed *before screening*:

Duplicate records removed

(n = 8,444)

**Identification**

Records excluded

(n = 17,740)

Records screened

(n = 17,847)

Reports not retrieved

(n = 0)

Reports sought for retrieval

(n = 107)

**Screening**

Reports assessed for eligibility

(n = 107)

Studies included in review

(n = 38)

Reports excluded (n = 69):

• Not related to health guidelines or HTAs (n = 40)

• No intervention impact on planetary health (n = 15)

• Does not provide advice on planetary health in guidelines/HTAs (n = 9)

• Not about planetary health (n = 5)

• Literature review without relevant advice (n = 4)

**Included**

# **Supplementary Appendix S4. List of included studies**

1.	Hensher M. Incorporating environmental impacts into the economic evaluation of health care systems: Perspectives from ecological economics. Resour Conserv Recycl. 2020 Mar 1;154:104623.

2.	Wu H, MacDonald GK, Galloway JN, Geng Y, Liu X, Zhang L, et al. A new dietary guideline balancing sustainability and nutrition for China’s rural and urban residents. iScience. 2022;25(10):105048.

3.	Wood A, Moberg E, Curi-Quinto K, Rysselberge P van, Roos E. From “good for people” to “good for people and planet” - placing health and environment on equal footing when developing food-based dietary guidelines. Food Policy. 2023;117.

4.	Wingrove K, Lawrence MA, Russell C, McNaughton SA. Evidence Use in the Development of the Australian Dietary Guidelines: A Qualitative Study. Nutrients. 2021 Nov;13(11):3748.

5.	White SM, Shelton CL, Gelb AW, Lawson C, McGain F, Muret J, et al. Principles of environmentally-sustainable anaesthesia: a global consensus statement from the World Federation of Societies of Anaesthesiologists. Anaesthesia. 2022;77(2):201–12.

6.	Walpole SC, Weeks L, Shah K, Cresswell K, Mesa-Melgarejo L, Robayo A, et al. How can environmental impacts be incorporated in health technology assessment, and how impactful would this be? Expert Rev Pharmacoecon Outcomes Res. 2023 Oct 21;23(9):975–80.

7.	Toolan M, Walpole S, Shah K, Kenny J, Jonsson P, Crabb N, et al. Environmental impact assessment in health technology assessment: principles, approaches, and challenges. Int J Technol Assess Health Care. 2023;39(1):e13.

8.	Tian J, McGrogan A, Jones MD. Low carbon footprint inhalers in England: a review of dispensing data. Int J Pharm Pract. 2022 Apr 1;30(Suppl 1):i38.

9.	Teschner R, Ruppen J, Bornemann B, Emmenegger R, Sánchez LA. Mapping Sustainable Diets: A Comparison of Sustainability References in Dietary Guidelines of Swiss Food Governance Actors. Sustainability. 2021 Jan;13(21):12076.

10.	Sharma M, Walpole S, Shah K. Spotlight environmental sustainability: a strategic priority for NICE. J Public Health Oxf Engl. 2022;44(4):e593–5.

11.	Serra-Majem L, Tomaino L, Dernini S, Berry EM, Lairon D, Ngo de la Cruz J, Bach-Faig A, Donini LM, Medina F-X, Belahsen R, et al. Updating the Mediterranean Diet Pyramid towards Sustainability: Focus on Environmental Concerns. International Journal of Environmental Research and Public Health. 2020; 17(23):8758.

12.	Seed B. Sustainability in the Qatar national dietary guidelines, among the first to incorporate sustainability principles. Public Health Nutr. 2015;18(13):2303–10.

13.	Schuitmaker-Warnaar TJ. ‘Green Metrics’ - Incorporating Environmental Dimensions In Health Technology Assessment: Health Technology Assessment international 2022 annual meeting. In 2022.

14.	Santaoja M, Jauho M. Institutional Ambiguity and Ontological Politics in Integrating Sustainability into Finnish Dietary Guidelines. Sustainability. 2020 Jan;12(13):5330.

15.	Rose D, Heller MC, Roberto CA. Position of the Society for Nutrition Education and Behavior: The Importance of Including Environmental Sustainability in Dietary Guidance. J Nutr Educ Behav. 2019;51(1):3–15.

16.	Rodriguez De Santiago E, Dinis-Ribeiro M, Pohl H, Agrawal D, Arvanitakis M, Baddeley R, et al. Reducing the environmental footprint of gastrointestinal endoscopy: European Society of Gastrointestinal Endoscopy (ESGE) and European Society of Gastroenterology and Endoscopy Nurses and Associates (ESGENA) Position Statement. Endoscopy. 2022;54(8):797–826.

17.	Ramjee L, Tremblay G, Forsythe A, Sandman K. PNS6 Saving Lives or the Environment - How about Both? the Role of Health Technology Assessment (HTA) Bodies in Creating Sustainable Healthcare Systems. Value Health. 2021;24(Supplement 1):S173–4.

18.	Polisena J, De Angelis G, Kaunelis D, Gutierrez-Ibarluzea I. Environmental impact assessment of a health technology: A scoping review. Int J Technol Assess Health Care. 2018;34(3):317–26.

19.	Ana-Catarina PG, Seo-Hyun Y, Alexander A, Hannah M, Koonal S, Michael T. Incorporating environmental and sustainability considerations into health technology assessment and clinical and public health guidelines: a scoping review. Int J Technol Assess Health Care. 2022;38(1):e84.

20.	Perreau S, Pauchard JC, Hafiani EM. Intégration progressive du développement durable par les systèmes de santé. Prat En Anesth Réanimation. 2021 Sep 1;25(4):167–74.

21.	Pekarsky BAK. The Inclusion of Comparative Environmental Impact in Health Technology Assessment: Practical Barriers and Unintended Consequences. Appl Health Econ Health Policy. 2020 Oct 1;18(5):597–9.

22.	Monteiro CA, Cannon G, Moubarac JC, Martins APB, Martins CA, Garzillo J, et al. Dietary guidelines to nourish humanity and the planet in the twenty-first century. A blueprint from Brazil. Public Health Nutr. 2015 Sep;18(13):2311–22.

23.	Miltenburger C, Borgstrom F, Eklund O. Ecological Benefits of Health Technologies – How to Consider Public Value in Funding Decisions? Value Health. 2018 Sep;21:S25.

24.	McGain F, Shelton C, McAlister S. French professional society guidelines on improving patient and planetary health: Liberté, égalité, fraternité… et durabilité. Anaesth Crit Care Pain Med. 2023 Oct 1;42(5):101292.

25.	McAlister S, Morton RL, Barratt A. Incorporating carbon into health care: adding carbon emissions to health technology assessments. Lancet Planet Health. 2022;6(12):e993–9.

26.	Martinez S, Delgado M del M, Marin RM, Alvarez S. Carbon footprint of school lunch menus adhering to the Spanish dietary guidelines. Carbon Manag. 2020 Jul 3;11(4):427–39.

27.	Kenny J, Shah K, Toolan M, Crabb N, Greaves F, Jonsson P, et al. Incorporating Environmental Impacts Into Health Technology Assessment: An Examination Of Potential Approaches And Challenges. Int J Technol Assess Health Care. 2022;38(Supplement 1):S29.

28.	Reynolds CJ, Buckley JD, Weinstein P, Boland J. Are the Dietary Guidelines for Meat, Fat, Fruit and Vegetable Consumption Appropriate for Environmental Sustainability? A Review of the Literature. Nutrients. 2014 Jun;6(6):2251–65.

29.	James-Martin G, Baird DL, Hendrie GA, Bogard J, Anastasiou K, Brooker PG, et al. Environmental sustainability in national food-based dietary guidelines: a global review. Lancet Planet Health. 2022 Dec 1;6(12):e977–86.

30.	Heuer R, Nast A. [Sustainable prescription and implementation practices in clinical practice guidelines]. Dermatol Heidelb Ger. 2023 Jan;74(1):34–40.

31.	Heuer R, Gaskins M, Werner RN, Nast A, Saha S. Planetary health in dermatology: towards a sustainable concept of health in clinical practice guidelines. Br J Dermatol. 2023 Jan 23;188(1):132–3.

32.	Herrmann A, Lenzer B, Müller BS, Danquah I, Nadeau KC, Muche-Borowski C, et al. Integrating planetary health into clinical guidelines to sustainably transform health care. Lancet Planet Health. 2022 Mar;6(3):e184–5.

33.	He P, Baiocchi G, Feng K, Hubacek K, Yu Y. Environmental impacts of dietary quality improvement in China. J Environ Manage. 2019 Jun 15;240:518–26.

34.	Guirado-Fuentes C, Abt-Sacks A, Trujillo-Martín M del M, García-Pérez L, Rodríguez-Rodríguez L, Carrion i Ribas C, et al. Main Challenges of Incorporating Environmental Impacts in the Economic Evaluation of Health Technology Assessment: A Scoping Review. Int J Environ Res Public Health. 2023 Jan;20(6):4949.

35.	González-García S, Green RF, Scheelbeek PF, Harris F, Dangour AD. Dietary recommendations in Spain –affordability and environmental sustainability? J Clean Prod. 2020 May 1;254:120125.

36.	Firth I, Hitch J, Henderson N, Cookson G. Moving towards a more environmentally sustainable pharmaceutical industry: recommendations for industry and the transition to green HTA. Expert Rev Pharmacoecon Outcomes Res. 2023;23(6):591–5.

37.	Dimmock P, Ali F, Wang Y, Islam L. PNS193 Capturing Environmental IMPACT in Health Technology Assessment of Medical Devices. Value Health. 2020;23(Supplement 2):S673.

38.	Aas AM, Axelsen M, Churuangsuk C, Hermansen K, Kendall CWC, Kahleova H, et al. Evidence-based European recommendations for the dietary management of diabetes. Diabetologia. 2023;66(6):965–85.

# **Supplementary Appendix S5. AGREE-II scores by domain**

|  | **Guideline** | | | |
| --- | --- | --- | --- | --- |
| **Domain** | Wu 2022 | White 2022 | Rodriguez DeSantiago 2022 | Aas 2023 |
| AGREE-2: Item 1 | 1 | 1 | 7 | 7 |
| AGREE-2: Item 2 | 1 | 1 | 7 | 4 |
| AGREE-2: Item 3 | 1 | 1 | 7 | 7 |
| AGREE-2: Item 4 | 1 | 4 | 4 | 7 |
| AGREE-2: Item 5 | 1 | 1 | 1 | 0 |
| AGREE-2: Item 6 | 1 | 7 | 7 | 7 |
| AGREE-2: Item 7 | 1 | 1 | 6 | 7 |
| AGREE-2: Item 8 | 1 | 1 | 7 | 3 |
| AGREE-2: Item 9 | 1 | 1 | 1 | 3 |
| AGREE-2: Item 10 | 1 | 7 | 7 | 7 |
| AGREE-2: Item 11 | 1 | 1 | 7 | 3 |
| AGREE-2: Item 12 | 1 | 1 | 4 | 3 |
| AGREE-2: Item 13 | 1 | 1 | 1 | 3 |
| AGREE-2: Item 14 | 1 | 3 | 1 | 7 |
| AGREE-2: Item 15 | 1 | 1 | 4 | 5 |
| AGREE-2: Item 16 | 1 | 1 | 1 | 7 |
| AGREE-2: Item 17 | 1 | 7 | 1 | 7 |
| AGREE-2: Item 18 | 1 | 6 | 7 | 7 |
| AGREE-2: Item 19 | 1 | 1 | 7 | 2 |
| AGREE-2: Item 20 | 1 | 4 | 6 | 1 |
| AGREE-2: Item 21 | 1 | 6 | 1 | 1 |
| AGREE-2: Item 22 | 1 | 7 | 1 | 6 |
| AGREE-2: Item 23 | 4 | 4 | 4 | 4 |
| AGREE-2: Domain 1: Scope and Purpose | 3 | 3 | 21 | 18 |
| AGREE-2: Domain 2: Stakeholder Involvement | 3 | 12 | 12 | 14 |
| AGREE-2: Domain 3: Rigour of Development | 8 | 16 | 34 | 36 |
| AGREE-2: Domain 4: Clarity of Presentation | 3 | 9 | 6 | 19 |
| AGREE-2: Domain 5: Applicability | 4 | 17 | 21 | 11 |
| AGREE-2: Domain 6: Editorial Independence | 5 | 11 | 5 | 10 |
| AGREE-2 Overall: 1 | 2 | 3 | 2 | 5 |
| AGREE-2: Recommended for Use | No | Yes | No | Yes |
| AGREE-2 Score Overall | 26 | 68 | 99 | 108 |
| AGREE-2 Percent | 16% | 42% | 61% | 67% |
